# Spectrophotometric Analysis of Coronal Discoloration In Vitro Induced by Bioceramic Cements

**DOI:** 10.3390/dj11070180

**Published:** 2023-07-24

**Authors:** Joana A. Carvalho, Carlos Franco, Luís Proença, João Albernaz Neves, Mário Polido, José João Mendes, Ana Mano Azul

**Affiliations:** 1 Egas Moniz School of Health and Science, Monte de Caparica, 2829-511 Almada, Portugal; joanaaraujo15423@gmail.com (J.A.C.); cfranco@egasmoniz.edu.pt (C.F.); lproenca@egasmoniz.edu.pt (L.P.); jalbernaz@egasmoniz.edu.pt (J.A.N.); mpolido@egasmoniz.edu.pt (M.P.); aazul@egasmoniz.edu.pt (A.M.A.); 2 Egas Moniz Center for Interdisciplinary Center (CiiEM), Monte de Caparica, 2829-511 Almada, Portugal

**Keywords:** bioceramics, spectrophotometric analysis, tooth discoloration

## Abstract

The aim of this study was to evaluate and quantify, in vitro, the extent of coronal discoloration induced by bioceramic materials over time. In total, 44 human monoradicular teeth were divided into four groups (*n* = 11), according to the applied material: Negative control (NC); White MTA ProRoot^®^ (WMTAP); Biodentine^™^ (BD); and TotalFill^®^ BC RRM^™^ Putty (TF). Teeth were immersed in saline solution and incubated at 37 °C; the following periods of time were defined: before incubation: [t0]—without bioceramic material, t1—immediately after the bioceramic material placement; after incubation: t24h—24 h, t7d—7 days, and 30d—30 days. Descriptive and inferential statistical analysis were performed. Cochran’s Q test was used to evaluate coronal discoloration across the different groups, while the Kruskal–Wallis test was employed to determine differences in discoloration among the groups at each time interval. Additionally, the Friedman test was applied to analyze the variations in discoloration within each group over time. A significance level of 5% was set. All experimental groups revealed coronal discoloration over time: NC (*p* = 0.001), WMTAP (*p* < 0.001), BD (*p* = 0.001), and TF (*p* = 0.006). No significant differences were observed between groups for each time interval (*p* > 0.05). The WMTAP group varied the most considering the intervals [t0—t1] and [t24h—t7d] (*p* = 0.037) compared to the remaining experimental groups.

## 1. Introduction

The dentin-pulp complex has the ability to respond to different aggressive stimuli, including caries lesions, mechanical trauma, and cavity preparation (occasionally iatrogenic), by promoting the deposition of hard tissue. This process is facilitated by the interaction of various constituents of the dentin matrix and may result in the differentiation of odontoblastic cells and the formation of tertiary dentin [1,2]. There are multiple techniques and procedures available for the treatment of pulp tissue in teeth with extensive caries lesions. In cases where the lesions are extensive and pulp exposure has occurred, dentists must consider different approaches. These include maintaining pulp vitality through the application of pulp protection materials and sealing the exposed dental pulp (direct pulp protection), removing part of the coronal pulp tissue (pulpotomy), or completely eliminating it (pulpectomy) [3].

In dentistry many devices have been introduced to designate more accurate colour selection, allowing us to provide better aesthetic results [4]. For colour measurement devices such as colorimeters, spectrophotometers, spectro-radiometers, or a combination of them can be used, which evaluate the amount and spectral composition of light that is reflected by the surface of the object to be measured. Generally, the results of these devices are expressed through the CIE L*a*b* system, which is used for the detection of all colours in the visible spectrum range through three coordinates [5].

Procedures related to vital pulpal therapy, including direct and indirect pulpal protection, pulpotomy, as well as techniques like stepwise excavation, perforation repair, and regenerative endodontics, often involve the placement of different types of materials, particularly in the coronal third of the tooth. However, it should be noted that such interventions may lead to coronal discoloration [6,7,8,9]. In specific cases involving patients with root resorption, perforations, or when procedures like apexification or retrograde obturation are necessary, new biocompatible materials have been developed, including bioceramic materials like MTA ProRoot^®^, Biodentine™, TotalFill^®^, Bioaggregate^®^, and Generex A^®^. These materials aim to enhance the patient’s prognosis and improve the final clinical outcome. Bioceramic materials are ceramic compounds that are derived through chemical processes, both in situ and in vivo. These materials offer notable advantages, such as remarkable biocompatibility, due to their composition that closely resembles hydroxyapatite. Additionally, they are non-toxic, non-shrinking, and typically exhibit chemical stability within the biological environment [10,11]. When in contact with bone tissue, these materials elicit a regenerative response, demonstrating an osteoconductive behavior. Mineral Trioxide Aggregate (MTA) holds the distinction of being the pioneering bioceramic material used in Endodontics and has consistently demonstrated positive outcomes [12].

In this present in vitro study, our objective is to evaluate and quantify the extent of coronal discoloration induced by various bioceramic materials and examine how this discoloration changes over time. The null hypothesis stated there are no significant differences in coronal discoloration over time when using different biomaterial cements.

## 2. Materials and Methods

### 2.1. Sample Preparation

For this laboratory study, which was approved by the Ethics Committee of Instituto Universitário Egas Moniz in 2017 (Protocol no. 544), a total of 44 permanent, sound human monoradicular teeth (central incisors, lateral incisors, canines and lower premolars), recently extracted, were obtained from the Human Tooth Bank at Egas Moniz Dental. Clinic. After scaling and cleaning, the teeth were stored in a 1% chloramine T solution at 4 °C for one week. Subsequently, they were transferred to distilled water, which was replaced weekly, and this process was continued until the teeth were utilized within six months of extraction.

The bioceramic materials are listed in Table 1.

### 2.2. Experimental Setup/Procedure

The 44 teeth were randomly assigned to four different groups (*n* = 11) based on the applied material using a computer random number generator. The groups were as follows: Negative Control (NC), White MTA ProRoot^®^ (WMTAP), Biodentine™ (BD), and TotalFill^®^ RRM™ Fast Set Putty (TF).

A radiovisiography (RVG) was conducted on each tooth using Kodak 6100 software. Then, the roots were cross-sectioned so the distance between the cemento–enamel junction to the sectioned site would be 10 mm.

The negative control consisted in a retrograde approach with instrumentation, irrigation, and subsequent filling of the tooth with only swine blood. Filtek Z250 composite resin (3M™ ESPE™, St. Paul, MN, USA) was used as a retrograde root filling.

For the other experimental groups, access cavities were prepared with a round #12 diamond bur.

All root canals were instrumented with R50 Reciproc files (VDW, Munich, Germany) and irrigated with 10 mL 3% sodium hypochlorite, 10 mL 17% EDTA and again 10 mL 3% sodium hypochlorite. All teeth were filled with swine blood and Filtek Z250 composite resin (3M^™^ ESPE^™^, St. Paul, MN, USA) was used as retrograde root filling. They were then distributed to each experimental group, and the specific bioceramic material was applied 3 to 4 mm apically to the cemento–enamel junction.

Resin-modified glass ionomer Vitrebond^™^ (3M^™^ ESPE^™^, St. Paul, MN, USA) was placed above the experimental material. Then, access cavities were then restored with an etch and rinse bonding strategy with 37% orthophosphoric acid (3M^™^ ESPE^™^, St. Paul, MN, USA) and Adper^™^ Scotchbond^™^ 1 XT Vial (3M^™^ ESPE^™^, St. Paul, MN, USA), according to the manufacturer’s instructions. Filtek Z250 composite resin (3M^™^ ESPE^™^ St. Paul, MN, USA) was used as the restorative material in a matching color with the tooth crown.

**Figure 1:**
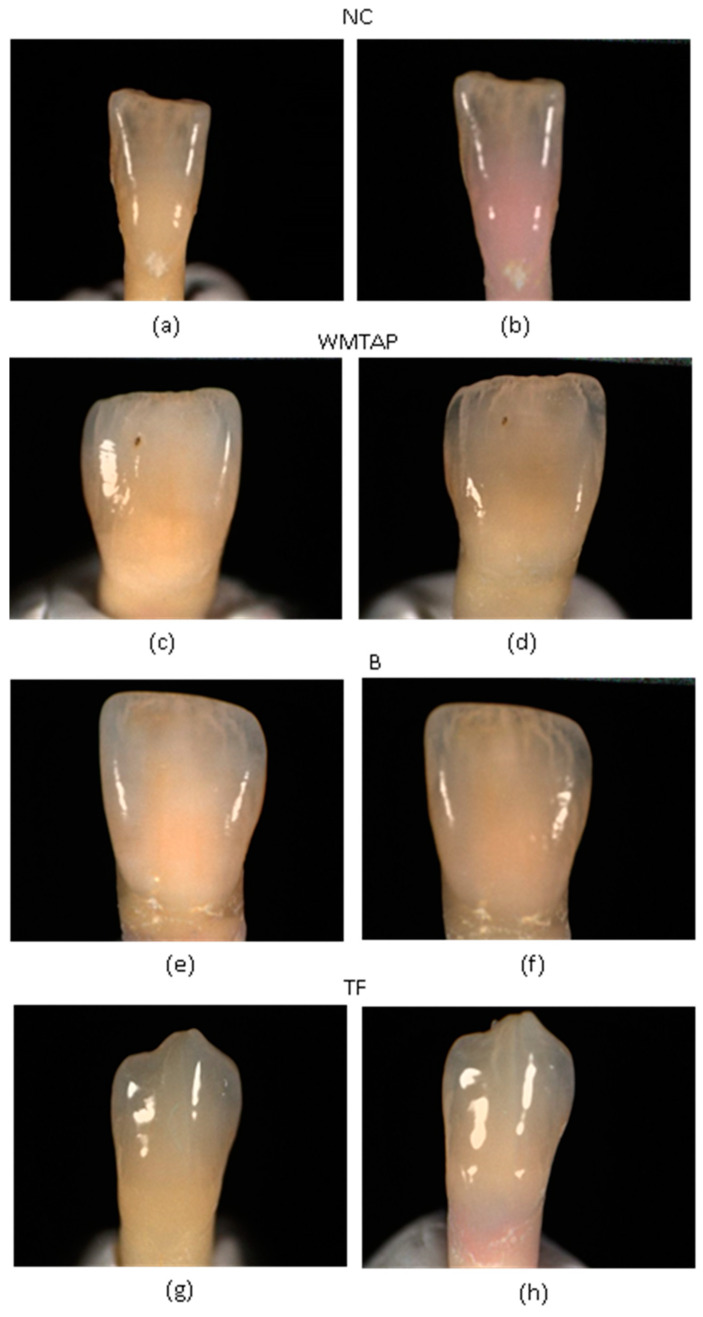
Illustrates an example of coronal discoloration observed at time t0 and t30d for the different groups. (**a**) NC-t0 (w/o swine’s blood); (**b**) NC-t30d (w/swine’s blood); (**c**) WMTAP-t0 (w/o WMTAP, and w/o incubation); (**d**) WMTAP-t30d (w/WMTAP and 30 days of incubation); (**e**) BD-t0 (w/o BD, and w/o incubation); (**f**) BD-t30d (w/BD and 30 days of incubation); (**g**) TF-t0 (w/o TF and w/o incubation; (**h**) TF-t30d (w/TF and 30 days of incubation).

After this procedure, the teeth were incubated (Memmert INE 400, Schwabach, Germany) at 37 °C with 100% humidity and immersed in 0,9% physiological saline with 0.5459 g of Chloride and 0.354 g Sodium (Thermo Fisher Scientific, Porto Salvo, Portugal) at pH 8.48. The following times were defined: t0—before incubation and without material placement; t1—before incubation and immediately after the experimental material placement; t24h—one day of incubation after placement of the experimental material; t7d—seven days of incubation after placement of the material; and t30d—thirty days of incubation after placement of the experimental material, with weekly renewal of the liquid in which they were stored (Figure 1).

The teeth’s color changes between the different moments of evaluation were obtained through the coordinates of the CIE L*a*b* system and ΔE formula (ΔE* = [(ΔL*)^2^ + (Δa*)^2^ + (Δb*)^2^], with the Spectroshade Micro Optic spectrophotometer (MHT S.p.A., Arbizzano di Negar, Italy), according to the manufacturer’s instructions and at the times previously described [4].

### 2.3. Statistical Analysis

Statistical Analysis was evaluated using the software Statistical Package for Social Sciences (SPSS) (IBM Corporation, Armonk, NY, USA) v. 26.0 and involved descriptive and inferential statistical measures. Given the characteristics of the variables to be evaluated, non-parametric tests were applied, since the assumptions for the application of parametric tests, after validation, were not verified.

The following tests were used: non-parametric Q Cochran test to determine whether there was coronal discoloration in the different experimental groups over time; Kruskal–Wallis to evaluate whether there were differences in discoloration in the four experimental groups for each time interval; and finally, the Friedman test to assess the variation of discoloration in the different groups. A ∆E ≥ 3.3, which translates to unacceptable discoloration easily detected by the naked eye, was taken into consideration to determine whether or not coronal discoloration existed [13,14]. A significance level of 5% was set.

## 3. Results

### 3.1. Descriptive Analysis

The descriptive analysis was performed for each of the coordinates of the CIE L*a*b* system including ΔE.

First of all, the L* parameter was analysed at time t0 and t30d. This parameter represents brightness and can range from black to white [15,16]. Table 2 shows that in all groups from t0 to t30d the L* value decreased, as well as ΔL that was negative for all groups, which means that the sample became darker throughout time Regarding a* parameter of the CIE L*a*b* system, it represents chroma in the axis that goes from red to green [15,16]. The WMTAP, BD, and NC groups showed a decrease in this parameter except for the TF group which showed an increase in a* (Table 3). The b* parameter of the CIE L*a*b* system, represents chroma on the axis from yellow to blue [15,16]. Table 4 shows that all groups showed a decrease in this parameter.

Based on the analysis presented in Table 5, it is evident that the ΔE* parameter for each experimental group varies over time. The observed discoloration in the given time intervals follows the order: TF > WMTAP > NC > BD.

### 3.2. Inferential Analysis

Cochran’s Q test was used to evaluate coronal discoloration across the different groups, while the Kruskal–Wallis test was employed to determine differences in discoloration among the groups at each time interval. Additionally, the Friedman test was applied to analyze the variations in discoloration within each group over time. A significance level of 5% was set.

Table 6 presents the frequency of discoloration over time. Initially (t0), no teeth exhibited discoloration; however, a gradual increase in the number of discolored teeth was observed at all time points within the sample of 44 teeth (*p* < 0.001-Cochran’s Q test). According to Ruyter, Nilner and Moiler, ΔE values equal or above 3.3 are visually perceptible and clinically unacceptable. Therefore, this threshold was considered for the determination of significant discoloration [13].

Subsequently, the presence of any differences in discoloration among the four experimental groups was examined for each evaluated interval. The probabilities of significance are presented in Table 7.

The distribution of ΔE values was identical among all experimental groups at each time point (t0–t1, t1–t24h, t24h–t7d, t7d–t30d) (*p* > 0.05-Kruskal–Wallis test).

**Figure 2:**
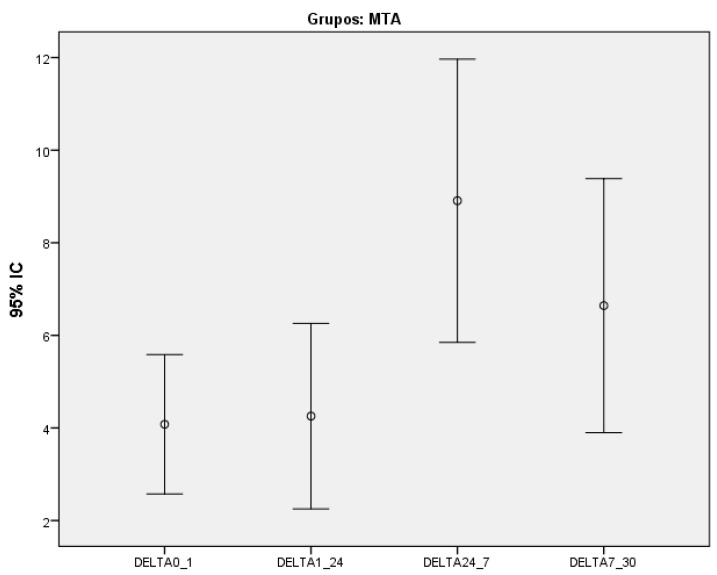
Variation of discoloration between the different time intervals for White MTA ProRoot^®^ group.

Furthermore, we assessed whether a significant change in discoloration had occurred within each group between the evaluated time intervals. The probabilities of significance are presented in Table 8. Only WMTAP showed a significant difference in discoloration between time intervals (*p* < 0.037-Friedman test) (Figure 2)

## 4. Discussion

Aesthetics hold a significant influence over our daily lives in today’s society. Numerous materials employed in endodontic procedures can result in visible discoloration of the tooth crown, which directly impacts an individual’s aesthetic appearance.

In this current in vitro study, our aim was to assess the extent of coronal discoloration caused by White MTA ProRoot^®^ (Dentsply, York, USA), Biodentine™ (Septodont, Saint-Maur-des-Fósses, France), and TotalFill^®^ BC RRM™ Putty (FKG, Crêt-du-Locle 4, CH 2304, La Chaux de Fonds, Switzerland), as well as to observe any changes in discoloration over a period of time. All groups exhibited significant discoloration over time (*p* < 0.001). Among the groups, the TF group showed the highest ΔE parameter in the last time interval [t7d–t30d], followed by the WMTAP group, then the NC group, and finally the BD group.

According to multiple studies, it has been observed that teeth tend to become discolored when the ΔE value reaches or exceeds 3.3. This discoloration is often perceived as aesthetically unpleasant and intolerable [13,14,17,18,19,20]. There was a variation in the number of discolored teeth except in the WMTAP group. However, for the BD, TF and NC groups, from time t0 to time t30d, there was a slight decrease in the number of teeth that previously showed discoloration. According to Beatty and Svec, this finding corresponds to the ‘rebound effect’ [16]. The NC group developed a diffuse pink-red discoloration on day 1, gradually increasing, and invading the coronal portion of the teeth. Regarding the WMTAP and TF groups, there was a clear grey discoloration that progressively intensified. Finally, in the BD group there was also a grey discoloration, but of lower intensity compared to the other groups. Although it was possible to identify higher and less levels of discoloration over time among the different groups, no significant differences were observed. Of all groups, the WMTAP group was the one that most varied in the time intervals [t0–t1] and [t24h–t7d]. The ΔL parameter, from time t0 to time t30d was always negative and there were no significant differences between the four experimental groups. However, the limits of 95% confidence intervals were always negative for the WMTAP and NC groups. Thus, in these two groups a considerable darkening at the crown level occurred [16]

One possible explanation for the discoloration observed in the control group is the potential infiltration of blood and its components, particularly red blood cells, into the dentinal tubules. This infiltration can result in hemolysis, leading to the release of hemoglobin. When mixed with necrotic pulp tissue, this process produces hydrogen sulfide, which ultimately causes a deep, dark grey or greyish-brown discoloration at the crown level [19,21,22]. Based on this observation, it appears that the bioceramic material in some teeth of this study was not sustained above the blood. When a tooth experiences trauma, be it iatrogenic, due to caries, or even mechanical causes, it can result in pulp hemorrhage. Consequently, there is a significant likelihood of subsequent coronal discoloration [23]. Pulp tissue necrosis, the presence of pulp remnants after endodontic treatment, specific materials used in endodontic procedures, and root resorption are among the intrinsic causes that can result in coronal discoloration [18]. When bismuth oxide comes into contact with sodium hypochlorite, a reaction takes place, resulting in a noticeable color change [24]. In a study conducted by Felman and Parashos (2013), it was found that wMTA contributed to coronal discoloration. Furthermore, the presence of blood constituents increased the likelihood of discoloration. However, more studies will be necessary to ascertain the factors that lead to coronal discoloration [25]. One possible reason why Biodentine™ shows comparatively less discoloration (although not statistically significant) than other bioceramic materials is its quick setting time, which effectively blocks the passage of blood to the remaining dental structures [23]. In a study conducted by Shokouhinejad et al. in 2015, it was observed that various bioceramic materials (MTA ProRoot, ERRM Putty, Biodentine, and Ortho MTA) exhibited an increase in discoloration when blood was applied [23,24,25,26]. In this study, it was noted that handling Biodentine™ material presented some challenges due to its fast-setting time. The bioceramic material that was found to be the easiest to place was TotalFill^®^ BC RRM™ Putty, followed by White MTA ProRoot^®^. It is important to address the limitations of the present study. The study tested coronal discoloration over time periods culminating in 30 days. Longer periods could be introduced to assess long-term color variation.

## 5. Conclusions

All experimental groups showed coronal discoloration over time, which was consistent among the different groups. This discoloration resulted from the use of swine blood and the application of bioceramic materials. Since various materials used in endodontic procedures can cause discoloration, it is important to inform patients about this potential occurrence. Further research is needed to advance the development of biomaterials in Endodontics that not only demonstrate effectiveness in treatment but also minimize discoloration.

## Figures and Tables

**Table 1:** Bioceramic cements and their specifications: White MTA ProRoot^®^, Biodentine^™^ and TotalFill^®^ RRM^™^ Fast Set Putty.

Commercial Name (Abbreviation)	Manufacturer	Lot	Expiration Date
White MTA ProRoot^®^	Denstply Tulsa Dental Products, Tulsa, OK, USA	000014787	12 August 2019
Biodentine^™^	Septodont Saint Maur-des Fósses, France	B19471	October 2018
TotalFill^®^ RRM^™^ Fast Set Putty (FKG)	Crêt-du-Locle 4 CH-2304 La Chaux-de-Fonds, Switzerland	1601FSPS	31 March 2018

**Table 2:** Mean values (M) of the parameter L and ΔL, standard deviations (SD), and 95% confidence intervals (CI) at t0 and t30d.

Groups	*N*	t0 (L*) M + SD (CI 95%)	t30d (L*) M + SD (CI 95%)	∆L*
NC	11	72.23 ± 3.70 [69.74; 74.71]	68.41 ± 2.93 [66.44; 70.38]	−3.82
WMTAP	11	74.90 ± 3.47 [72,57; 77,23]	71,95 ± 1.94 [70.65; 73.26]	−2.95
BD	11	71.64 ± 3.24 [69.46; 73.81]	69.73 ± 3.12 [67.63; 71.82]	−1.91
TF	11	72,20 ± 3,73 [69.69; 74.71]	71.35 ± 4.40 [68.39; 74.30]	−0.85
Total	44			

**Table 3:** Mean values (M) of a* parameter and Δa*, respective standard deviation (SD), and 95% confidence interval (CI) at t0 and t30d.

Groups	*N*	t0 (a*) M + SD (IC 95%)	t30d (a*) M + SD (IC 95%)	∆a*
NC	11	1.30 ± 2.18 [−0.17; 2.77]	1.28 ± 1.98 [−0.06; 2.61]	−0.02
WMTAP	11	1.31 ± 1.85 [0.07; 2.55]	0.72 ± 1.23 [−0.11; 1.54]	−0.59
BD	11	2.17 ± 1.56 [1.13; 3.21]	1.66 ± 1.14 [0.89; 2.43]	−0.51
TF	11	1.38 ± 2.15 [−0.06; 2.83]	1.65 ± 2.17 [0.19; 3.10]	0.27

**Table 4:** Mean values (M) of parameter b* and Δb*, respective standard deviation (SD), and 95% confidence interval (CI) at t0 and t30d.

Groups	*N*	t0 (b*) M + SD (IC 95%)	t30d (b*) M + SD (IC 95%)	∆b*
NC	11	19.49 ± 4.24 [16.64; 22.34]	14.11 ± 1.97 [12.79; 15.43]	−5.38
WMTAP	11	19.50 ± 3.32 [17.27; 21.73]	16.94 ± 3.62 [14.50; 19.37]	−2.56
BD	11	21.60 ± 4.47 [18.60; 24.60]	18.70 ± 5.72 [14.86; 22.54]	−2.90
TF	11	19.49 ± 4.24 [16.64; 22.34]	18.06 ± 4.08 [15.33; 20.80]	−1.43
Total	44			

**Table 5:** Mean values (M) of the ΔE* parameter respective standard deviation (SD), and 95% confidence interval (CI) in the following time intervals [t0–t1], [t1–t24h], [t24h–7d], and [7d–30d].

Groups	*N*	∆E 0–1 M + SD (CI 95%)	∆E 1–24h M + SD (CI 95%)	∆E 24h–7d M + SD (CI 95%)	∆E 7d–30d M + SD (CI 95%)
NC	11	9.00 ± 4.73 [5.82; 12.18]	6.18 ± 6.72 [1.67; 10.70]	7.36 ± 4.05 [4.64; 10.08]	6.40 ± 2.59 [4.65; 8.14]
WMTAP	11	4.08 ± 2.24 [2.57; 5.58]	4.26 ± 2.98 [2.25; 6.26]	8.91 ± 4.55 [5.85; 11.97]	6.64 ± 4.08 [2.73; 10.41]
BD	11	7.44 ± 3.82 [4.88; 10.01]	5.36 ± 3.65 [2.91; 7.81]	6.57 ± 5.71 [2.73; 10.41]	5.49 ± 4.48 [2.48; 8.50]
TF	11	7.42 ± 5.63 [3.64; 11.20]	3.20 ± 1.64 [2.10; 4.30]	7.12 ± 5.34 [3.53; 10.70]	7.29 ± 5.99 [3.26; 11.32]
Total	44				

**Table 6:** Discoloration frequency (ΔE* ≥ 3.3) over time.

Discoloration Frequency						
Groups	t0	t1	t24h	t7d	t30d	*p*
NC	0	9	5	10	9	<0.001 *
WMTAP	0	6	6	10	10	<0.001 *
BD	0	10	8	7	7	0.001 *
TF	0	8	5	7	6	0.006 *
Total	0	33	24	34	32	<0.001 *

* Significant *p*-values (*p* < 0.05).

**Table 7:** Probability of significance (*p*) of discoloration (ΔE* ≥ 3.3) between experimental groups over time.

Groups	N	∆E 0–1 *p*	∆E 1–24h *p*	∆E 24h–7d *p*	∆E 7d–30d *p*
NC	11	0.075	0.779	0.611	0.779
WMTAP	11				
BD	11				
TF	11				
Total	44				

**Table 8:** Probability of significance (*p*) of discoloration (ΔE ≥ 3.3) for the different experimental groups over time.

Groups	*p*
NC	0.409
WMTAP	0.037 *
BD	0.315
TF	0.210

* Significant *p*-values (*p* < 0.05).

## Data Availability

Data are available upon request from the corresponding author.
